# Impaired innate and conditioned social behavior in adult C57Bl6/J mice prenatally exposed to chlorpyrifos

**DOI:** 10.1186/s12993-019-0153-3

**Published:** 2019-03-01

**Authors:** Anat Lan, Daniel Stein, Miguel Portillo, Debra Toiber, Ora Kofman

**Affiliations:** 1 Department of Psychology, Ben-Gurion University of the Negev, POB 653, Beer-Sheva, Israel; 2 Department of Life Sciences, Ben-Gurion University of the Negev, POB 653, Beer-Sheva, Israel; 3 Zlotowski Center for Neuroscience, Ben-Gurion University of the Negev, POB 653, Beer-Sheva, Israel

**Keywords:** Chlorpyrifos, Social behavior, Prenatal exposure, Organophosphate pesticides, Oxytocin

## Abstract

**Background:**

Signs of pervasive developmental disorder and social deficits were reported in toddlers and children whose mothers were exposed to organophosphate pesticides during pregnancy. Deficits in social preference were reported in adult male mice exposed to chlorpyrifos on gestational days 12–15. This study aimed (a) to test the hypothesis that adult female and male mice that were exposed prenatally to subtoxic doses of chlorpyrifos would be impaired in social behavior and (b) to determine if prenatal chlorpyrifos altered the expression of transcripts for oxytocin in the hypothalamus. Pregnant mice were treated by gavage with corn oil vehicle or 2.5 mg/kg or 5 mg/kg of CPF on gestational days 12–15. Social preference, social and non-social conditioned place preference tasks were tested in adults. Expression of oxytocin transcripts in hypothalamus was measured by qPCR.

**Results:**

Chlorpyrifos (5 mg/kg on GD 12–15) reduced the innate preference for a conspecific in a dose and sex dependent manner. Adult males exposed prenatally to 5 mg/kg CPF showed a reduction in social preference. Socially conditioned place preference was impaired in offspring of dams treated with either dose of CPF. Non-social appetitive place conditioning was impaired in offspring of dams exposed to 2.5 mg/kg, but not to 5 mg/kg chlorpyrifos. Prenatal chlorpyrifos treatment did not alter the expression of the oxytocin mRNA in the hypothalamus, although expression was significantly lower in females.

**Conclusions:**

Prenatal chlorpyrifos induced innate and learned social deficits and non-specific conditioning deficits in adult mice in a sex-dependent manner. Males showed specific social deficits following the higher dose whereas both males and females showed a more generalized conditioning deficit following the intermediate dose.

## Background

The role of environmental pollutants, including organophosphate pesticides (OP) such as chlorpyrifos (CPF), as a vulnerability factor in the etiology of autism spectrum disorders (ASD) has garnered attention of researchers [26] given that heritability for autism is incomplete [18]. In support of a link between pesticide exposure and ASD, the Childhood Autism Risks from Genetics and Environment (CHARGE) study found a positive association between ASD and residential proximity to OP application during the second and third trimester of gestation in a population-based case control study in California [47]. In addition to ASD, deficits in cognition and attention were associated with residential proximity to OP use during gestation in the Center for the Health Assessment of Mothers and Children of Salinas (CHAMACOS) Study in the Salinas Valley, California [17]. The level of OP metabolites in maternal urine during pregnancy was associated with deficits in social reciprocal behavior, which were more prominent in boys than girls in the Mount Sinai Children’s Environmental Health Center study [14]. However, a recent study on the association between maternal exposure to OPs and signs indicative of ASD was equivocal [46]. In two major longitudinal studies in the USA, the CHAMACOS project [13] and the Columbia project in New York [43], symptoms of pervasive developmental disorder (PDD) in toddlers were reported in offspring of mothers who showed evidence of significant exposure to organophosphate pesticides (OP) during gestation. Prior to the publication of the fifth edition of the Diagnostics and Statistical Manual in Mental Disorders (DSM V) [1], PDD was the term used for socialization and communication deficits that did not meet full criteria for autism.

The possibility of OP exposure as a risk factor for ASD is not limited to agricultural environments, as OPs are ubiquitous in food, dust, and air. It was estimated that children under age 5 potentially inhale 2 ng/kg/day and potentially ingest close to 5 ng/kg/day in their home and daycare environments [20, 29, 33, 55]. Moreover, exposure to pesticides from environmental contact in infants and toddlers exceeds that of adults because the former have a higher metabolic rate and more frequent hand-to-mouth contact [27].

### Effects of exposure to OPs during gestation on infant and child development

In North America [13, 30, 55, 56] and Asia [24, 53], an association was found between the level of organophosphate dialkylphosphate (DAP) metabolites in pregnant mothers’ urine or in neonate cord blood and lower birth weight. Reviews of the studies on the long-term effects of maternal exposure to pesticides during gestation have yielded conflicting conclusions, supporting [19] or opposing [31] the view that CPF exposure has deleterious effects on infant development. The former review was based on a meta-analysis of longitudinal studies after stratification of the birth and development scores by ethnic origin [19]. There was a negative relation between DAP metabolites and birth weight in blacks and between DAP and gestational length in whites. In addition to weight, delayed development of neonatal reflexes [11, 56] and lower scores on the Bayley Mental Development Index (MDI) at 12 and 24 months and on the Psychomotor Development Index at 36 months [43] were associated with higher maternal exposure to OPs. OPs are rapidly metabolized such that maternal urinary diethylphosphate (DEP) or dimethylphosphate (DMP) metabolites are not a direct reflection of foetal exposure; however, these levels were associated with residential proximity to pesticide use in the CHAMACOS cohort [4]. In the Columbia University longitudinal studies, CPF exposure was also derived from monitoring maternal air exposure, maternal blood and neonate cord blood. As described above, these measures were related to changes in psychomotor and mental development [42, 54].

Higher order cognitive deficits were observed in children who had been exposed to OPs during gestation. Attention deficits were reported at ages 36 months and 5 years [30, 43]. Lower IQ scores and impaired working memory were reported in different longitudinal studies at 7 years [3, 12, 42]. In contrast, a more recent study found a positive association between maternal DMP during pregnancy and maternal-reported measures of executive function in children aged 6–9 [15].

Studies focusing on sex differences in the effects of gestational OP exposure have led to inconclusive results [7]. On one hand boys showed greater deficits than girls on social [14] and working memory tests [21] in correlation with gestational exposure to chlorpyrifos (CPF); however in a sample of children genetically at high-risk for ASD, OP exposure was more likely to be associated with ASD in the girls than the boys [38]. Since the effects of prenatal OPs on behavioral outcomes have not been adequately explored in the pre-clinical literature, in the current study, both male and female mice were assessed for the effects of gestational CPF on social behavior.

### Long-term effects of prenatal exposure to CPF on rodent behavior

Prenatal exposure to CPF resulted in delayed motor development, impaired innate social preference and conditioned social preference, and restricted interest in adult male mice [25], behaviors used to model symptoms of ASD in preclinical studies [34, 44]. While delayed reflexes are not specifically related to the autism spectrum, delays in reaching motor milestones, abnormal reflexes, and postural asymmetries were reported to be prevalent in infants and toddlers who were later diagnosed with ASD [2].

Exposure to CPF during late gestation enhanced offensive posture, but not attacks, in adult male mice [45] and increased social discrimination in adult female mice [9]. Dietary CPF exposure during gestation extending to postnatal day 14 led to enhanced social investigation of familiar conspecifics in female mice and both familiar and novel mice in males [52]. In contrast, gestational CPF had no effect on investigation of a conspecific in adult female BTBR mice, which have been used as a model for autism, possibly because of their low baseline level of social preference [8]. Exposure of wild type Reelin positive mice to CPF oxon from embryonic day 13.5 through weaning also did not affect social preference in male or female adult offspring at postnatal day 30 [35].

Notably, many of the assays that have been used to assess the long-term effects of gestational CPF exposure on social behavior involve an element of learning, raising the question of whether the behavioral change is an indirect consequence of a more general deficit or advantage induced by CPF. For example, increased discrimination or investigation of familiar mice by female mice exposed to CPF suggests that the CPF-treated female mice had an advantage in social learning assays [9, 52]. Thus, although our main aim in the present study was to explore both innate (social preference) and conditioned (conditioned social place preference) social behaviors, we addressed the possibility that a more general change in learning would be found.

### Oxytocin involvement in the long-term effects of prenatal CPF

Although the lethal effect of CPF is attributed to inhibition of acetylcholinesterase (AChE), sub-toxic doses equal to or greater than those used in our studies produced transient or no significant inhibition of brain AChE in pups [45, 52] with brain AChE being less inhibited than serum AChE. Fetuses treated with 5 mg/kg CPF/day during mid or late gestation did not show significant changes in forebrain or brainstem markers for development at gestational days 17 or 21 [39]; however, long-term changes in forebrain integrity and cholinergic markers were observed postnatally [40]. In addition to long-term effects on cholinergic markers, other neurotransmitter systems and expression of a multitude of genes have been reported following gestational CPF [5, 22, 32, 48].

In the current study, we tested whether the expression of oxytocin (OT) transcripts was altered in the hypothalamus as reported previously [49]. OT is a peptide that mediates social behavior. OT antagonists attenuated social learning [10, 23], and social recognition was impaired in rodents lacking OT receptors [50]. Conversely, OT agonists facilitated social behavior, and potentiated prosocial behaviors induced by 3,4-methylenedioxy-*N*-methylamphetamine (MDMA) [10, 41].

The current study examined innate and learned social behavior in male and female adult mice exposed during gestation to CPF. A non-social control experiment was used to examine the specificity of the deficit, and the expression of mRNA for OT in the hypothalamus was assayed as a putative mechanism for the reported deficits.

## Results

### Social preference (SP)

**Fig. 1:**
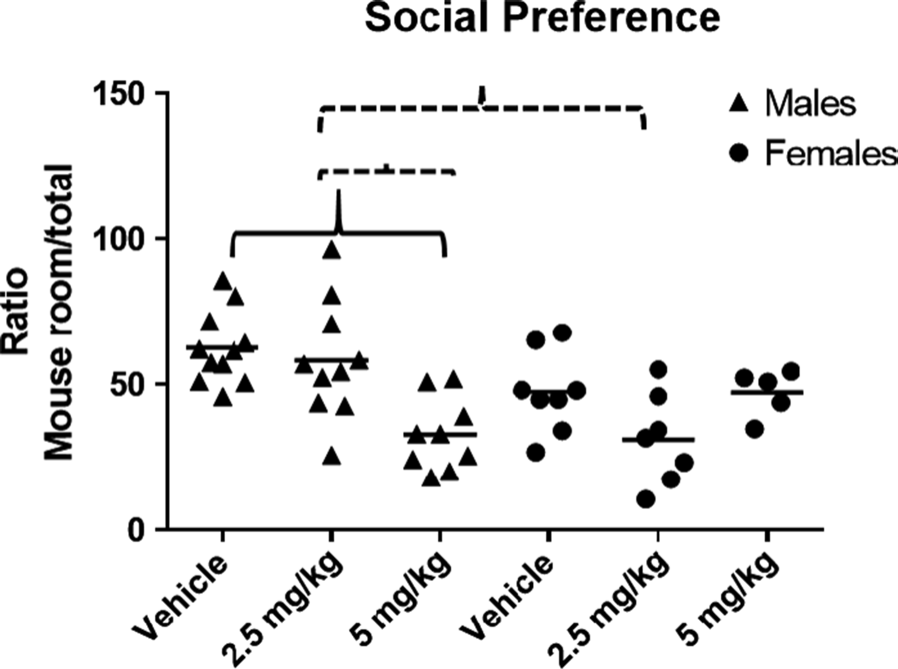
Social preference test. Mean preference for a same sex conspecific. Individual mice are shown as symbols (Δ male O female). All mice are offspring of dams age 90 days, which were given CPF (2.5 or 5 mg/kg) or peanut oil vehicle by gavage on GD 12–15. Social preference is calculated as the ratio of the time spent in the chamber containing the mouse to the total time spent in the apparatus, measured in seconds. Group differences: Solid line male p < .001; dashed line p = .007. Male vehicle: n = 11, male 2.5 mg/kg: n = 10, male 5 mg/kg: n = 9, female vehicle: n = 8; female 2.5 mg/kg: n = 7; female 5 mg/kg: n = 5

In the SP task, significant main effects for treatment, F (2, 44) = 4.32, p = .02, and sex were found, F(1,44) = 4.61, p = .04. The Levene’s test for homogeneity of variance was not significant (F = .75, p = .59). There was also a significant sex × treatment interaction, F(2,44) = 7.33, p = .002. The Tukey post hoc test revealed that male offspring of dams that had been treated with 5 mg/kg CPF had significantly lower SP than the male offspring whose dams had been treated with vehicle (p < .001) or 2.5 mg/kg CPF (p = .007). There were no group differences among female mice exposed prenatally to CPF or vehicle. The female offspring of dams treated with 2.5 mg/kg CPF had lower SP than the male offspring treated with 2.5 mg/kg CPF (p = .007). In conclusion, the male, but not the female mice exposed to CPF showed significantly less SP than the offspring of the vehicle-treated dams, replicating our previous result [25] and suggesting that CPF exposure during gestation elicits a deficit in social behavior (Fig. 1).

### Social conditioned place preference (SCPP)

The change in preference for the initially non-preferred bedding prior to and following social conditioning was measured. A change in the positive direction indicated that the conditioned stimulus, the bedding, acquired a positive value after being associated with social living conditions (the unconditioned stimulus). The Levene test for homogeneity of variance was significant for the SCPP test, F = 2.62, p = .04, leading to the decision to analyze the data with non-parametric statistics. The Kruskal–Wallis test for the treatment effect indicated that there was a significant effect of prenatal CPF exposure, H = 10.29, p = .006, with the control group, who were offspring of dams treated with the oil vehicle, ranking 32.18, compared to the offspring of mice treated with 2.5 mg/kg CPF (16.36) or 5.0 mg/kg CPF (21.39). The effect of sex was also statistically significant, H = 5.97, p = .015. These analyses showed that the offspring of mice treated with CPF did not acquire the socially conditioned bedding preference, in contrast to the offspring of vehicle-treated mice, and that female mice did not learn this conditioning as well as males.

### Food conditioned place preference (FCPP)

**Fig. 2:**
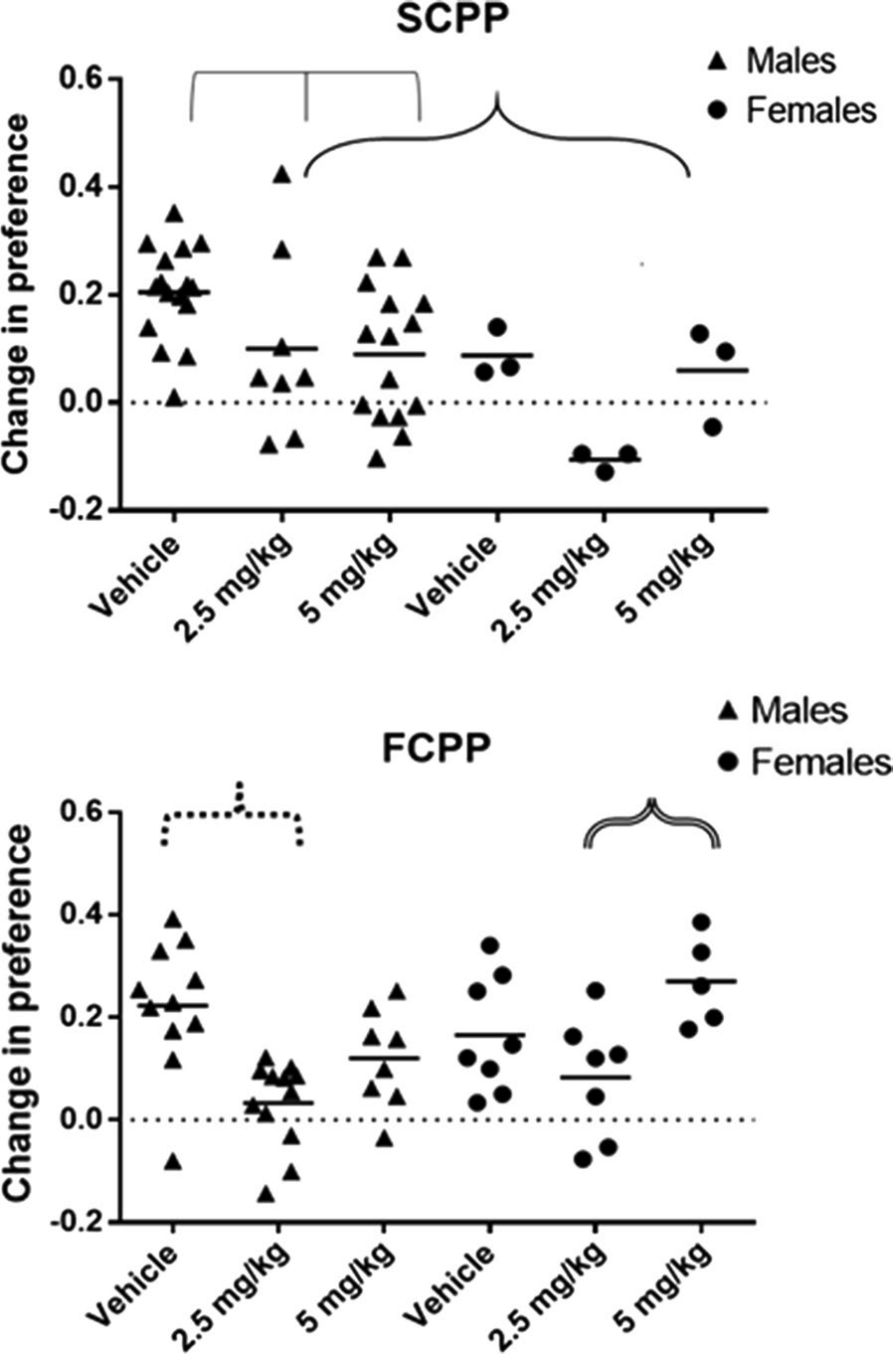
Top panel: social conditioned place preference test. Mean preference for the bedding that was conditioned with another conspecific. Individual mice are shown as symbols (Δ male O female). All mice are offspring, aged 90 days, of dams that were given CPF (2.5 or 5 mg/kg) or peanut oil vehicle by gavage on GD 12–15. The main effect of treatment is depicted by the thin triple bracket, indicating that the vehicle exposed group showed a larger change in preference than each of the CPF-exposed groups (p = .006). The thick bracket indicates the main effect of sex (p = .015). Male: vehicle: n = 16, 2.5 mg/kg: n = 8, 5 mg/kg: n = 15, female: n = 3 in each group. Bottom panel: food conditioned place preference test. Mean preference for the bedding that was conditioned with an appetitive stimulus reward. Individual mice are shown as symbols (Δ male O female). All mice are offspring aged 90 days of dams, that were given CPF (2.5 or 5 mg/kg) or peanut oil vehicle by gavage on GD 12–15. The significant comparisons are depicted by dashed brackets (p = .001) or double bracket (p < .05). Male: vehicle: n = 11, 2.5 mg/kg: n = 8, 5 mg/kg: n = 12, female: vehicle: n = 8, 2.5 mg/kg: n = 7. 5 mg/kg: n = 5

The change in preference for the initially non-preferred bedding following food conditioning was measured. Because the experiment was designed as a biased place preference, two mice were removed from the analysis because they did not show a preference for either bedding (one female offspring of a dam treated with vehicle and one female offspring of a dam treated with 5 mg/kg CPF). A positive change in value indicated that the mice learned to associate the initially non-preferred bedding with a positive appetitive reward and to increase their preference for that bedding. The Levene’s test for homogeneity of variance was not significant, F = .39, p = .84. A significant main effect for treatment was found, F(2,45) = 9.24, p = .0004, and a significant sex × treatment interaction F(2,45) = 3.55, p = .04. The male mice that were offspring of dams that had been treated with 2.5 mg/kg CPF on GD 12–15 dose showed less robust conditioning on the FCPP (Tukey’s test p < .001), compared to the male offspring of the vehicle treated group, whereas there was no significant appetitive conditioning deficit in the male offspring of dams treated with 5 mg/kg CPF. The female offspring of dams that had been treated with 2.5 mg/kg CPF on GD 12–15 dose showed an FCPP deficit compared to the female offspring of the dams treated with 5 mg/kg CPF p = .046, but they did not differ from the offspring of dams treated with vehicle (Fig. 2). These findings indicate that prenatal exposure to CPF reduced the ability of mice to learn to associate a cue with a non-social reward.

### OT transcripts

**Fig. 3:**
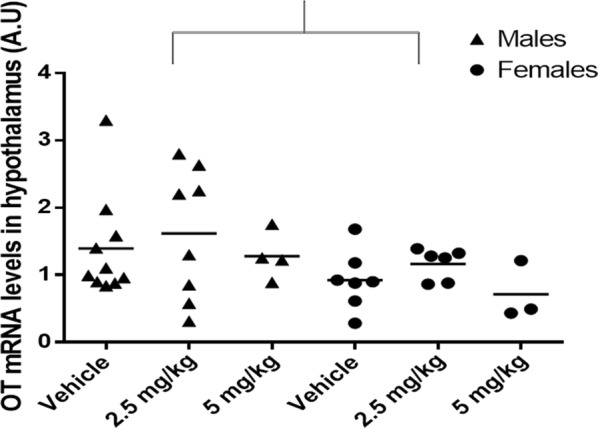
Quantitative PCR for oxytocin mRNA levels in the hypothalamus of adult offspring of mothers exposed to CPF on GD 12–15 (normalized to GAPDH mRNA levels). Male: vehicle: n = 10, 2.5 mg/kg: n = 8, 5 mg/kg: n = 4, female: vehicle: n = 7, 2.5 mg/kg: n = 6. 5 mg/kg: n = 3. The bracket indicates the main effect of sex, p < .05

CPF administration did not change the expression of the OT mRNA in the hypothalamus. After removing two outliers, whose values were more than three standard deviations above mean, an ANOVA was conducted for the effect of treatment and sex. There was no significant main effect for treatment, F (2,32) = .92, p = .41, nor a sex × treatment interaction, F(2,32) = .02, p = .98. However, a main effect for sex was found, F (1,32) = 4.51, p = .04. Females expressed less OT in the hypothalamus compared to males (Fig. 3).

## Discussion

In addition to replicating our previous findings that prenatal CPF induced deficits in the SP and SCPP task in males [25], the current study revealed both social and non-social conditioning deficits in both male and female mice after prenatal exposure to CPF, commensurate with the suggestion that exposure to pesticides during mid to late gestation can reveal sex-specific effects [7]. Administration of CPF on GD 12–15, at doses below the threshold for observable signs of toxicity, elicited sex-selective deficits in social behavior and caused a reduced ability to establish conditioning in the FCPP task. The main difference between the sexes was the fact that the female mice were not impaired in the SP test, an assay of inherent sociability that is commonly used to investigate social deficits in genetic models of ASD [34]. Male offspring of the dams that had been treated with 5 mg/kg CPF showed a deficit in both innate and learned social preference tests. These findings are in accord with findings that boys who had been exposed to OPs during gestation had more social deficits than girls [14]. In addition to the social deficits, offspring of dams that had been treated with 2.5 mg/kg CPF showed a deficit in the non-social appetitive conditioning task, the FCPP.

While the sociability deficit in males was consistent, the female offspring of dams that had been gavaged with CPF did not show a deficit in the social preference test even thought they were impaired in the SCPP. It is difficult to draw conclusions regarding social behavior of female offspring in this study for several reasons: (a) in general female mice showed a weak social preference whether innate or learned and (b) the number of females in the studies was very low in some groups (3–5), reducing the power of the analysis. De Felice et al. [9] pointed out that female mice exposed to gestational CPF showed more social discrimination, while gestational exposure to CPF had no effect in a strain with inherently low social behavior, the BTBR mice [8]. Venerosi et al. [51] found that 6 mg/kg exposure to CPF on GD 15–18 in outbred CD-1 mice led to more frequent social vocalization and investigation, an effect that was eliminated if the mice continued to be exposed to CPF postnatally. Mullen et al. [35] also found that adult female, but not male mice, which had been exposed during late gestation to CPF oxon, showed more social investigation than vehicle-treated mice. Thus, a range of effects have been found in female mice, but there is no conclusive evidence of a sociability deficit following exposure to CPF or its oxon metabolite.

Furthermore, this study revealed a more general conditioning deficit in both sexes which was evident in the offspring of mice exposed to the 2.5 mg/kg dose but not 5.0 mg/kg CPF. Although the source of this non-linear dose response is not clear, a similar phenomenon was reported by Levin et al. [28] who showed that a dose of 1 mg/kg of CPF on GD 17–20 induced more deleterious effects, compared to a dose of 5 mg/kg in females when working and reference memory in the radial-arm maze were tested in adult offspring in rats. A tentative hypothesis could involve the possible effect of CPF on increasing stimulus salience and discriminability, as pointed out by De Felice et al. [9] and in a test of fear conditioning in mice treated with CPF on post-natal days 4–10 [37]. In that study, the mice treated with CPF showed better discrimination between the conditioned stimulus and the interstimulus interval than the control mice. Thus, we speculate that the low dose of CPF may impair conditioning, but that this effect is countered by the enhanced cue salience in mice that were exposed to the higher dose of CPF. However, further research addressing the effects of perinatal CPF on cue salience and conditioning would be required.

The current study is in accord with some of the studies on humans that reported more severe cognitive and social deficits in boys who had been exposed to gestational OPs [14, 21]; however, there is no evidence for a specific social deficit in female offspring.

Although sex differences were not reported in the SP test, per se, estrogen alpha and beta mRNA expression were correlated with social interaction in regions associated with social recognition, such as amygdala, dorsal septum and medial preoptic area [6]. Estrogen replacement has both prosocial or asocial effects, depending on context [16]. Further investigation of the effects of gestational exposure to CPF should more closely control for hormonal influences by monitoring the hormonal state of the female.

Prenatal exposure to CPF did not affect the OT gene expression in the hypothalamus. This contrasted with increased OT peptide expression in the hypothalamus in males reported in adult mice that had been exposed to 6 mg/kg of CPF on GD 15–18 [49]. Another study by the same group showed that CPF administration on GD 15 to lactation day 14 did not change the expression of OT in the hypothalamus, but decreased the expression of OT in the amygdala in males [52]. Together these data suggest that exposure on different periods of development may result in different alternations of brain function, an issue which requires further research.

Exposure to CPF during the second trimester of gestation led to impaired social preference and conditioned social preference in male, but not female adult offspring. However, conditioning impairments in the FCPP suggest that the CPF exposure impairs both social and non-social learning for positive reinforcement. There was no effect of CPF on the expression of OT transcripts in the hypothalamus; however further studies are required to examine the effects on OT and its receptor in brain areas relevant to reward conditioning.

## Conclusions

Exposure to low dose pesticides is a global health problem which can impair brain development in utero. In the present study, prenatal CPF induced innate and learned social deficits and non-specific conditioning deficits in adult mice in a sex-dependent manner. Male offspring of dams treated with 5 mg/kg CPF showed specific social deficits, whereas both males and female offspring of dams treated with 2.5 mg/kg CPF showed a more generalized conditioning deficit. The mechanism for this deficit requires further investigation, as in this study, we did not find a relation between the behavioral findings and hypothalamic oxytocin.

## Materials and methods

### Mice breeding and prenatal treatment

All experiments were conducted on C57BL/6 (B6) mice, using one naive male and one naïve female mouse per litter for each of the behavioral assessments and another male and female from the same litter for the OT gene and receptor expression evaluation. Dams and sires for breeding were purchased from Harlan, Israel. Animals were housed in polycarbonate breeding cages in a temperature controlled environment (22 ± 1 °C) under a 12-h reversed light–dark cycle (21:00–9:00 lights on) and ad libitum food **(**SSNIFF V1154-703 10 mm gamma irradiated 25 kGy) and water. After mating two females to one male, females were examined daily for the presence of vaginal plug, designating gestational day (GD) 0. On GD 12, the dams were randomly assigned to one of three prenatal treatments: high (5 mg/kg) or low dose of CPF (2.5 mg/kg), or corn oil (vehicle). Dams were housed individually after the plug was discovered and then housed with their litter until weaning. CPF (99.5% purity, Chem Service, Inc.) or corn oil (Willi Food, Yavneh, Israel) was administered by gavage to pregnant females daily from GD 12 to 15 in a volume of 0.1 ml/10 g body weight using a 22 gauge stainless steel feeding tube (Solomon Instech, Inc.).

Pups were weaned at 28 days and housed in same sex littermate cages of 2–5 pups. The doses that were selected did not induce overt cholinergic symptoms in dams, such as tremor or lacrimation.

All the tests were performed blindly by having a research assistant or investigator remove the identities of the treatment groups and number the cages consecutively. The code was broken only when the data were analyzed. On each testing day, mice from each group was tested. The order of testing was counterbalanced. The protocols were approved by the Institutional Committee for the Ethical Care and Use of the Animals in Experimentation of Ben-Gurion University of the Negev.

### Social preference

At 90 days of age innate social preference was tested on one female and one male per litter [25]. The testing box (24 × 16 cm) was divided into 3 chambers (8 × 16 cm each). The middle chamber (8 × 16 cm) that had a small opening on either side to allow the mouse to pass freely between chambers. Each side chamber contained an overturned perforated oval plastic cup (10.4 × 9.4 cm × 12.2 cm high), one of which enclosed an unfamiliar, untreated conspecific of the same age and sex. The perforations allowed nose to nose contact but prevented other direct contact. This apparatus is similar to that used in the classical studies of social preference and social novelty to test genetic mouse models of autism [34, 36]. Mice serving as social stimuli were habituated to the apparatus the day before testing by confining them to the container for 10 min. The experimental mouse was placed in the middle chamber and allowed to freely explore the 3 chambers for 10 min without the object or mouse and then tested the following day for 10 min in the presence of the empty container or the container under which the stimulus mouse was confined. The sides containing the social stimulus vs empty container were counterbalanced among mice. The time spent and number of entries into the chamber containing the stimulus mouse and that with the empty container were measured offline by an experimenter blind to the treatment condition using Ethovision™ software. Social preference was calculated as the ratio of the time spent in the chamber containing the mouse to the total time spent in the apparatus (measured in seconds).

### Social conditioned place preference (SCPP)

First the preferred bedding of each mouse was tested in a preference test similar to the SP described in the previous section, except that instead of choosing between a cup concealing a conspecific and an empty cup, in this test the mouse had to choose between two novel types of bedding, either aspen sawdust or shredded paper. Pilot data on mice that did not participate in this study indicated that there was no overall preference for either type of bedding, but that individual mice tended to show a preference for one or the other bedding. A negatively biased paradigm was used in order to reverse the initial preference by associating the non-preferred bedding to social reward. Hence, the non-preferred bedding served as the conditioned stimulus to be associated with the social living condition, whereas the initially preferred bedding was associated with the isolation condition. Conditioning was accomplished by housing the experimental mouse with its non-preferred bedding and a cage mate for 24 h followed by 24 h housing alone with the initially preferred bedding. The housing conditions were alternated for a total of 10 days with food and water available *ad lib.* The conditioned preference was assessed for each mouse as described for social preference. Each side contained a cupful of one of the two beddings (social and isolation) spread on the floor of the chamber. The day before the test the experimental mouse was allowed to freely explore the chamber for 10 min in the absence of sawdust and on the test day they were place in the middle chamber and allowed to explore the 3 chambered apparatus in the presence of the two bedding stimuli. The sides of the positively or negatively conditioned beddings were counterbalanced among the mice. Time spent in each chamber, measured in seconds, was scored via the Ethovision software by an observer blind to the treatment condition.

### Food conditioned place preference (FCPP)

A deficit in SCPP could indicate either a social deficit or a more general impairment in contextual conditioning. In order to test the specificity of the deficit, we tested the mouse’s ability to learn contextual conditioning to a specific bedding that was associated with a non-social appetitive cue (soup croutons, Osem Ltd.). During the conditioning phase, each mouse was housed individually in a cage with either the novel aspen or shredded paper bedding. Regular chow was restricted to the equivalent of 9% of body weight in grams, such that a mouse weighing 30 g, received 2.7 g chow each day. Water was given *ad lib*. For the positive conditioned stimulus, 10 croutons were hidden in the bedding each day, whereas nothing was hidden in the bedding that was not associated with reward (the stimulus that predicted no reward). The habituation and preference testing occurred as described for the SCPP. A change in the bedding baseline preference was measured as an indication for conditioning the bedding stimulus to the food treat. Data were analyzed by an observer blind to the treatment condition using the Ethovision software.

### Oxytocin transcript analysis

On PND 90, naïve mice that were littermates of those tested in the behavioral studies were sacrificed and the hypothalamus was removed when the ventral surface was exposed. In order to isolate OT RNA we homogenized brain tissues using Precellys Lysing Kit (Ref. KT03961-1-993.2). Then the tissues were run for 30 s twice in a homogenizer (maximum speed) (Minilys^®^, Bertin Instruments) and RNA was isolated using Biological Industries EZ-RNA II kit, following its protocol. For reverse transcription, we used QIAGEN QuantiTect Reverse Transcription Kit (Ref. 205311), using the recommended kit protocol. We reverse-transcribed 200 ng RNA to cDNA. Finally, for quantitative PCR (qPCR) we used BioRad SsoAdvanced Universal SYBR Green Supermix (Cat. No. 172-5271) kit and BioRad CFX384 machine, following the kit protocol:

1. 95 °C, 5 min
2. 95 °C, 15 s
3. 60 °C, 30 s
4. Repeat steps 2–3 for 39 more times
5. Melting curve: 65–95 °C, .5 °C increments, 5 s/step.

Each qPCR reaction final volume was 12 μl, with cDNA equivalent to 10 ng RNA, and primer concentrations of 300 nM each. Each combination of a sample and a primer set ran in triplicates. Primer sequences for OT were: Forward-GAC CTG GAT ATG CGC AAG TGT and reverse-GAA GCA GCC CAG CTC GTC. The sequences of the ‘housekeeping’ gene glyceraldehyde 3-phosphate dehydrogenase (GAPDH) were: Forward-AGA GAC AGC CGC ATC TTC TTG and reverse-GTA ACC AGG CGT CCG ATA CG.

### Statistical analysis

Before analyzing the data using analysis of variance (ANOVA) the Levene’s test for homogeneity of variance was performed. If the assumption of homogeneity of variance was rejected, the data were analyzed by the non-parametric Kruskal–Wallis test. This was done in the case of Social Conditioned Place Preference. If the Levene’s test was not significant, data were analyzed by two-way analysis of variance (ANOVA) for the effect of prenatal treatment (vehicle, 2.5 or 5 mg/kg) × sex (male, female) for each of the dependent variables. If there was a significant effect of treatment or a significant interaction involving treatment, a post hoc Tukey test was performed.
